# Antinociceptive Effect of Hydantoin 3-Phenyl-5-(4-ethylphenyl)-imidazolidine-2,4-dione in Mice

**DOI:** 10.3390/molecules20010974

**Published:** 2015-01-08

**Authors:** Ronaldo Bezerra de Queiroz, Fabíola Lélis de Carvalho, Diogo Vilar da Fonsêca, José Maria Barbosa-Filho, Paula Regina Rodrigues Salgado, Luciano Leite Paulo, Ana Bárbara Maroja de Queiroz, Liana Clébia de Morais Pordeus, Severino Araújo de Souza, Helivaldo Diogenes da Silva Souza, Bruno Freitas Lira, Petrônio Filgueiras de Athayde-Filho

**Affiliations:** 1 Center for Health Sciences, Federal University of Paraiba, João Pessoa PB 58059-900, Brazil; E-Mails: qronaldo@ig.com.br (R.B.Q.); fabiolalelis@ltf.ufpb.br (F.L.C.); divilar@hotmail.com (D.V.F.); jbarbosa@ltf.ufpb.br (J.M.B.-F.); paulasalgado87@gmail.com (P.R.R.S.); luciano-sjp@hotmail.com (L.L.P.); bmqueiroz1@gmail.com (A.B.M.Q.); liana@ccs.ufpb.br (L.C.M.P.); 2 Department of Chemistry, Federal University of Paraiba, João Pessoa PB 58059-900, Brazil; E-Mails: sasindustrial@bol.com.br (S.A.S.); helivaldog3@gmail.com (H.D.S.S.); brunofrlira@hotmail.com (B.F.L.)

**Keywords:** hydantoin, psychopharmacology, antinociceptive, anti-inflammatory

## Abstract

Imidazolidine derivatives, or hydantoins, are synthetic compounds with different therapeutic applications. Many imidazolidine derivatives have psychopharmacological properties, such as phenytoin, famous for its anticonvulsant efficacy, but also effective in the treatment of neuropathic pain. The hydantoin, 3-phenyl-5-(4-ethylphenyl)-imidazolidine-2,4-dione (IM-3), synthesized from the amino acid, glycine, was selected for psychopharmacological studies in mice on the basis of its chemical and structural similarity with phenytoin. The first step of this study was to define the LD_50_, which determined the doses of 50, 100 and 200 mg/kg for subsequent tests. The results obtained from the behavioral screening indicated that IM-3 produces decreased ambulation and analgesia in mice. Motor coordination and anxiety behavior were not affected by treatment with IM-3, as observed in the rotarod and elevated plus-maze tests, respectively. Regarding its antinociceptive properties, IM-3 showed efficacy in the acetic acid-induced writhing test by increasing the latency of the first writhe and reducing the number of writhes, as well as reducing the paw licking time in the second phase of the formalin test. The behavior of treated animals exposed to the hot plate test, however, did not differ from that of the control group. These data suggest that IM-3 has antinociceptive effects in mice, which is probably mediated by anti-inflammatory mechanisms.

## 1. Introduction

Amino acids are the building blocks of receptors, enzymes, peptide hormones and some neurotransmitters and, thus, play important roles in the body. Even glycine, the simplest amino acid, is essential to the normal functioning of the central nervous system (CNS), where it works as one of the most important inhibitory neurotransmitters and is known to modulate pain perception [1,2].

Pain perception is related to the way in which the same noxious stimulus is experienced differently when applied to different individuals [3]. It is determined not only by the amount of nociceptive input, but mainly by descending modulatory mechanisms, which involve inhibitory and facilitatory pathways from the CNS [4]. This process is influenced by the individual characteristics of each person and includes age, ethnicity, gender, past experiences, memories and emotional state, among others [5,6,7,8].

Since glycine is directly involved in the pain modulation process, the importance of screening for drugs obtained from this amino acid as potential tools in the management of pain is clear, especially when considering the side effects of the therapeutic options currently available or even with their lack of efficacy in some clinical conditions [9].

Several studies [10,11,12] have described the synthesis of amino acid compounds, their importance and applications as intermediates for the synthesis of heterocyclics [13,14]. The present study aimed to contribute to the pharmacological studies of imidazolidine compounds obtained from amino acids. Accordingly, the hydantoins, heterocyclic structures obtained by organic synthesis from glycine and characterized by the imidazolidine-2,4-dione ring, are considered potential drugs for the treatment of pain. The attention focused on this class is also noted by the clinical efficacy of phenytoin, one of its main representatives, as an ally in the treatment of neuropathic and cancer pain [15,16]. Despite its extensive use, phenytoin has a narrow therapeutic index, which requires attention. Toxicological effects include nystagmus, loss of smooth pursuit and eventually coma [17]. Given these disadvantages, the development of a new improved drug is important to reduce such problems.

As other studies have demonstrated the efficacy of some hydantoin derivatives in animal pain models [18,19], this study investigated the psychopharmacological activity of the hydantoin derivative, 3-phenyl-5-(4-ethylphenyl)-imidazolidine-2,4-dione (IM-3), in mice, particularly its antinociceptive activity.

## 2. Results and Discussion

To obtain the target compound, the initial strategy was to synthesize *C*-4-ethylphenylglycine from 4-ethylbenzaldehyde, which reacts with sodium cyanide and ammonium chloride (Strecker synthesis), followed by acid hydrolysis to form the IM-3 compound, according to our published procedure [20].

### 2.1. Acute Toxicity

IM-3 at doses of 400 and 800 mg/kg did not cause mortality in mice. In the groups treated with 1200 and 2000 mg/kg IM-3, 10% and 70% death was observed, respectively, while in the group that received 3000 mg/kg, there was 100% lethality. These results made it possible to calculate by nonlinear regression the LD_50_ of IM-3 in mice by the intraperitoneal route, which was estimated to be 1358.9 mg/kg, with a confidence interval of 1000–1584.9 mg/kg.

### 2.2. Behavioral Pharmacological Screening

At a dose of 50 mg/kg, increased grooming was observed after 240 min of observation, with no change in the preceding minutes. At a dose of 100 mg/kg, no alteration was observed compared to the control group, except an increase in grooming behavior at 120 min. Treatment with 200 mg/kg IM-3 resulted in analgesia in response to clamping the tail up to 180 min of observation. Also noticed was reduced constipation and ambulation in the first 60 min and increased grooming behavior from 120 to 240 min of observation.

### 2.3. Rotarod Test

As shown in Table 1, treatment with 50, 100 and 200 mg/kg IM-3 did not affect the motor performance of mice subjected to the rotarod test at 30, 60 or 120 min after administration.

**Table 1:** Effect of 3-phenyl-5-(4-ethylphenyl)-imidazolidine-2,4-dione (IM-3) on the rotarod test in mice.

Treatment	Dose (mg/Kg, i.p)	Time Spent on the Rotarod (Minutes)		
		30	60	120
Control (10 mL/kg)	-	179.3 ± 0.7	167.8 ± 11.2	173.0 ± 6.0
IM-3	50	136.4 ± 19.7	162.4 ± 15.5	161.4 ± 18.6
	100	133.9 ± 18.9	159.4 ± 18.8	177.9 ± 1.4
	200	146.9 ± 18.0	163.6 ± 15.2	179.4 ± 0.6

Data are expressed as the mean ± SEM. (*n* = 8). ANOVA followed by Dunnett’s test.

### 2.4. Elevated Plus-Maze Test

None of the doses of IM-3 tested (50, 100 and 200 mg/kg) were able to change the number of entries or the time spent in open arms (Table 2).

**Table 2:** Effect of IM-3 on the elevated plus-maze test in mice.

Treatment	Dose (mg/kg, i.p.)	Open Arms	
		Number of Entries	Duration (s)
Control (10 mL/kg)	-	2.5 ± 0.8	41.2 ± 14.3
IM-3	50	3.2 ± 0.6	41.5 ± 14.3
	100	2.2 ± 0.8	22.0 ± 9.5
Diazepam	0.5	6.4 ± 0.6 **	90.7 ± 14.2 **

Data are expressed as the mean ± SEM. (*n* = 8). ANOVA followed by Dunnett’s test. ** *p* < 0.01.

### 2.5. Acetic Acid-Induced Writhing Test

**Figure 1:**
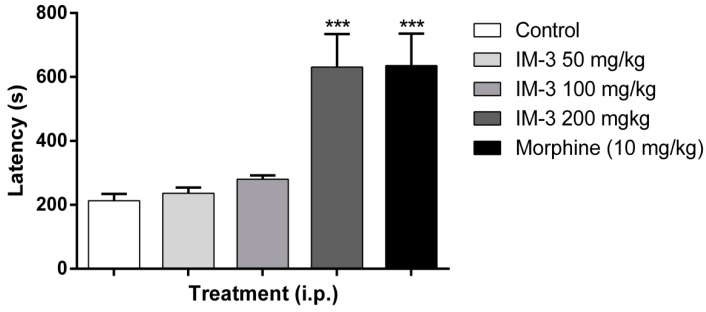
Effect of IM-3 on the latency of the first contortion in the acetic acid-induced writhing test in mice (*n* = 8). ANOVA followed by Dunnett’s test. *** *p* < 0.001.

**Figure 2:**
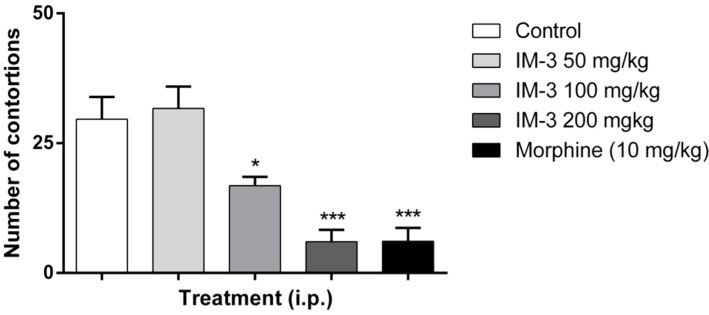
Effect of IM-3 on the number of contortions in the acetic acid-induced writhing test in mice (*n* = 8). ANOVA followed by Dunnett’s test. * *p* < 0.05, *** *p* < 0.001.

As shown in Figure 1, only IM-3 at 200 mg/kg (630.7 ± 103.5 s) was able to significantly increase the latency of the first contortion compared to the control group (213.3 ± 20.9 s). Figure 2 shows the effects of IM-3 at doses of 50, 100 and 200 mg/kg i.p. on the number of writhes in mice. At doses of 100 and 200 mg/kg, there was a significant decrease in the number of writhes (16.8 ± 1.7 and 6.0 ± 2.3 writhes, respectively) compared to the control group (29.6 ± 4.3 writhes).

### 2.6. Formalin Test

**Figure 3:**
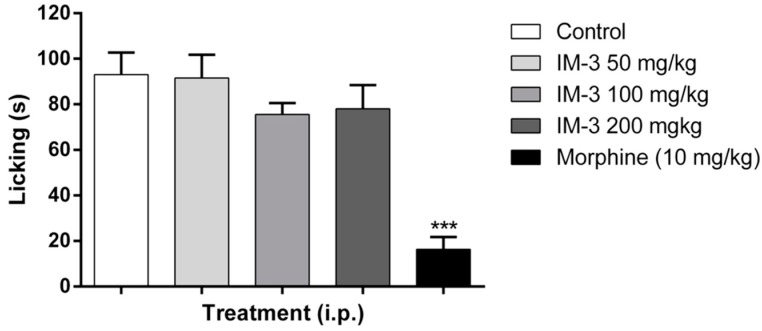
Effect of IM-3 on the first phase of the formalin test in mice (*n* = 8). ANOVA followed by Dunnett’s test. *** *p* < 0.001.

**Figure 4:**
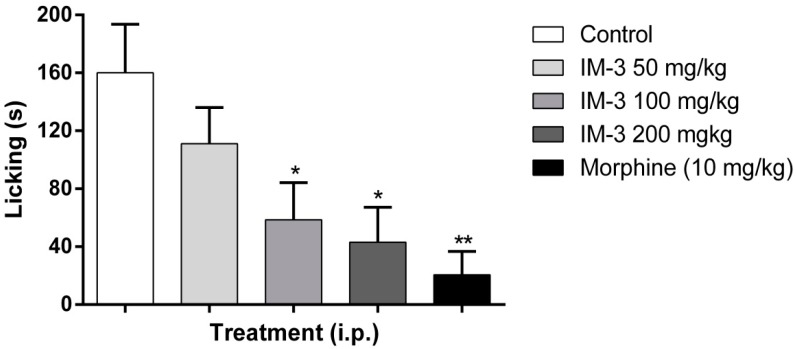
Effect of IM-3 on the second phase of the formalin test in mice (*n* = 8). ANOVA followed by Dunnett’s test. * *p* < 0.05; ** *p* < 0.01.

IM-3, at the doses tested (50 mg/kg: 91.6 ± 10.2 s; 100 mg/kg: 75.5 ± 5.0 s; 200 mg/kg: 78.0 ± 10.4 s), showed no significant reduction in paw licking time in the first phase of the formalin test when compared to the control group (93.0 ± 9.8 s) (Figure 3). According to the results presented in Figure 4, IM-3 decreased the paw licking time in the second phase of the formalin test at doses of 100 and 200 mg/kg (58.5 ± 25.7 and 43.1 ± 24.2 s, respectively), when compared to the control group (160.1 ± 33.4 s). Animals treated with morphine showed decreased paw licking time in both phases (16.3 ± 5.4 s in the first phase and 20.6 ± 16.3 s in the second phase).

### 2.7. Hot Plate Test

**Figure 5:**
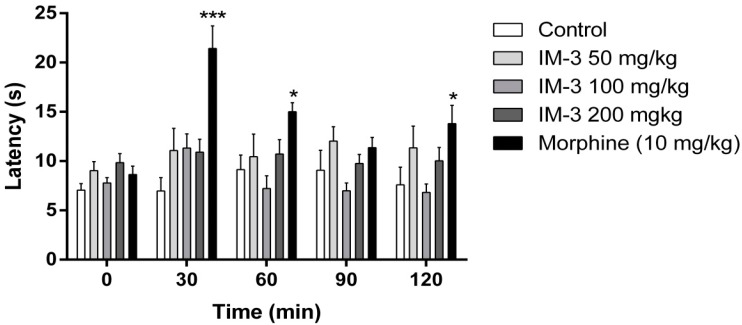
Effect of IM-3 on the second phase of the formalin test in mice (*n* = 8). ANOVA followed by Dunnett’s test. * *p* < 0.05; ** *p* < 0.001.

Figure 5 shows the effects of IM-3 at doses of 50, 100 and 200 mg/kg i.p. on the time mice spent on a hot plate. There was no indication of an antinociceptive effect of IM-3 at the doses tested with regard to this parameter when compared to the control group.

### 2.8. Discussion

This study investigated the antinociceptive activity of 3-phenyl-5-(4-ethylphenyl)-imidazolidine-2,4-dione (IM-3) in mice using behavioral methods.

The determination of LD_50_ was helpful to estimate the toxicity of IM-3 and to establish the nontoxic doses that could be used, thus avoiding the possibility of toxic effects being confused with the true psychopharmacological effects of IM-3. The LD_50_ of IM-3 by the i.p. route was 1358.9 mg/kg, with a confidence interval of 1000–1584.9 mg/kg. Based on this result, doses of 50, 100 and 200 mg/kg were chosen for the following behavioral experiments.

Behavioral psychopharmacological screening is a preliminary test of easy execution and able to detect in a qualitative way some important central actions [21]. The doses of IM-3 tested caused decreased ambulation and analgesia. These behavioral alterations are similar to those exhibited by substances that lower CNS activity. The decrease in ambulation in mice may be the most common way of determining if the test substance has depressor effects on the CNS, but it is imprudent to use this single parameter to classify a drug. A reduction in the response to a mechanical stimulus is observed after treatment with analgesic substances, and the presence of this behavior after administration of new drugs could be an indication of antinociceptive activity [22].

Despite the depressant effect observed in behavioral screening, IM-3 did not impair the motor coordination of mice, as observed in the rotarod test. Similar results have been reported for the hydantoin derivatives, 5-phenylmethylenehydantoin (5-PMH), and its para-methoxy substituted derivative (5-*p*-MPMH), while the ortho- and meta-substituted analogues (5-*o*-MPMH and 5-*m*-MPMH, respectively) significantly reduce the ability of the mice to remain on the rotarod [23], demonstrating the relevant role of the substituent groups on the effects of these drugs.

The performance of mice on the elevated plus-maze test was not affected by the treatment with IM-3, indicating a lack of anxiolytic effect.

Luis *et al.* (2010) [20] synthesized a series of hydantoins and affirmed that, when given to mice at doses of 250 and 500 mg/kg i.p., IM-3 reduced the number of writhes in the acetic acid-induced writhing test, but did not alter the latency in the hot plate test. The current study complements and extends the findings obtained by these authors on the antinociceptive activity of IM-3 using lower doses.

The acetic acid-induced writhing test was chosen to confirm the antinociceptive effects of IM-3 evidenced at behavioral psychopharmacological screening. This test is a simple, fast and reliable model to estimate the antinociceptive activity of substances. This test is sensitive to both central and peripheral drugs [24,25], as intraperitoneal administration of acetic acid induces the release of prostaglandins and CNS mediators [26,27].

IM-3 was found to reduce, in a dose-dependent manner, the number of abdominal contractions, as demonstrated previously at higher doses [20], as well as increasing the latency of the first writhe at the highest dose tested, when compared to the control group.

The formalin test is a chemical model of nociception that provides a greater specific response compared to the acetic acid model [28]. It is considered a model of clinical pain [29], since two different phases of nociception can be evaluated. The first phase begins immediately after formalin injection and lasts for the first 5 min (neurogenic or acute pain), where it is related to direct chemical stimulation of A δ fibers (Aδ-) and C-fiber nociceptors, and the release of excitatory amino acids, nitric oxide and P substance. The second phase occurs between 15 and 30 min after formalin, and it is related to the release of many pro-inflammatory mediators, such as bradykinin, prostaglandins and serotonin [30]. Drugs that act at the central level, such as opioid analgesics, inhibit both phases of the formalin test; however, drugs with peripheral action, such as anti-inflammatory ones, are effective only during the second phase.

None of the doses of IM-3 tested (50, 100 and 200 mg/kg) decreased the paw licking time during the first phase of the formalin test, unlike morphine and other central acting drugs. Doses of 100 and 200 mg/kg reduced paw licking time during the second phase, which is indicative of the anti-inflammatory activity of IM-3. On the other hand, the hydantoins, 5-(1*H*-indol-3-yl-methylene)-2-thioxo-imidazolidin-4-one and 3-(4-bromo-benzyl)-5-(1*H*-indol-3-yl-methylene)-2-thioxo-imidazolidin-4-one, studied by Guerra *et al.* (2011) [18], reduced the paw licking time in both phases of the formalin test in mice.

To investigate the involvement of IM-3 in central structures, the hot plate test was performed, which is a selective test that uses heat as a nociceptive stimulus to identify analgesic substances with a central effect [31].

The doses of IM-3 tested (50, 100, 200 mg/kg) did not alter the latency of the thermal stimulus response in the hot plate test, confirming the results obtained by Luis *et al.* (2010) [20]. Sudo *et al.* (2010) [32], in turn, observed that the hydantoin derivative, PT-31 (3-(2-chloro-6-fluorobenzyl)-imidazolidine-2,4-dione), probably exerts its antinociceptive response via central mechanisms, since it increased the nociceptive threshold in mice subjected to the hot plate challenge after intraperitoneal administration. The two indole-imidazolidine derivatives (LPSF/NN-52 and LPSF/NN-56) studied by Guerra *et al.* (2011) [18] did not alter the latency response in the hot plate test in mice, just like IM-3. These authors also demonstrated that the mechanism of action of both compounds is probably not associated with a central action, but rather with their ability to reduce the levels of pro-inflammatory cytokines, such as TNF-α and IL-1, and decrease leukocyte migration.

## 3. Experimental Section

### 3.1. Animals

Swiss albino male mice (*Mus musculus*), aged 2 to 3 months old, weighing 25 to 35 g, were used. The animals were housed in polyethylene cages and maintained under controlled temperature (21 ± 1 °C), with a 12-hour light/dark cycle (lights on at 6:00 a.m.), with food (Purina^®^ commercial pellet feed, Paulínia-SP, Brazil) and water available *ad libitum* until 60 min prior to tests.

Mice were placed in polyethylene cages with 4 animals each at least 60 min before tests, to minimize the eventual behavioral alterations of the animal. Each animal was used only once and euthanized at the end of the test. All experimental procedures were analyzed and previously approved by the Ethics Committee on Animal Use of Federal University of Paraiba, under Certificate No. 0404/10.

### 3.2. Drugs and Treatments

**Figure 6:**
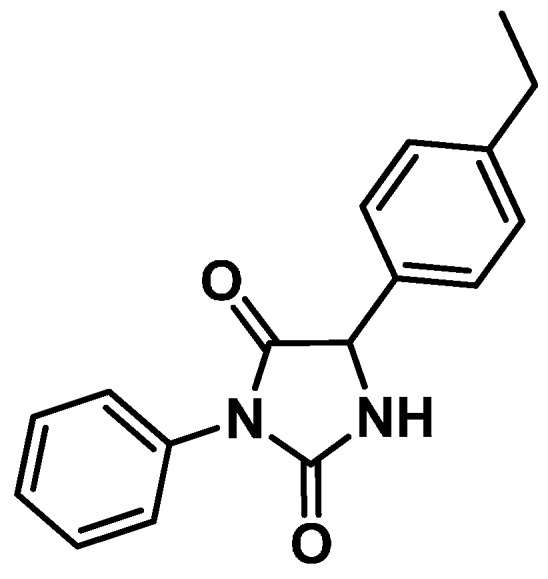
Chemical structure of hydantoin derivative IM-3 (3-phenyl-5-(4-ethylphenyl)-imidazolidine-2,4-dione).

The synthetic compound, 3-phenyl-5-(4-ethylphenyl)-imidazolidine-2,4-dione (IM-3), shown in Figure 6, was provided by the Laboratory of Research in Bioenergy and Organic Synthesis (LPBS) and obtained as follows. Amino acid 4-ethylphenylglycine (2.0 g, 10 mmol) was dissolved with mixing in 35 mL of 10% sodium hydroxide. After dissolution, phenyl isocyanate (1.19 g, 10 mmol) was slowly added with mixing. The solution was further mixed for 4 h and then acidified with HCl (12N) until a precipitate formed. The precipitate was filtered and refluxed for 2 h in 100 mL of 6 N HCl. The precipitate obtained was filtered and washed with ethanol and air dried. Recrystallization with distilled water/ethanol (1:1) resulted in 2.87 g (90.0% yield) of white crystals (melting point: 216–218 °C). ^1^H-NMR (DMSO-*d*_6_, TMS) δ: 1.16 (t, 3H, C*H*_3_CH_2_ *J* = 7.6 Hz); 2.60 (q, 2H, CH_3_C*H*_2_ *J* = 7.6 Hz); 5.33 (s, 1H); 7.24–7.51 (m, 9H, aromatics); 8.97 (s, 1H, NH) ppm. ^13^C-NMR (DMSO-d6,TMS) δ: 15.7 (*C*H_3_CH_2_); 27.9 (CH_3_*C*H_2_); 59.8; 126.8; 127.1; 127.9; 128.2; 128.8; 132.2; 133.1; 144.3; 155.8; 171.9 (C=O) ppm (for the experimental details, see our published procedure [20]).

Minutes prior to its utilization, IM-3 was dissolved in 3.4% Tween 80 in distilled water. The control group received the vehicle used for IM-3 solution (3.4% Tween 80). Morphine (Sigma, São Paulo-SP, Brazil) was used as the standard drug in positive control groups and was diluted in distilled water. All drugs and the vehicle were administered intraperitoneally (i.p.) in a volume of 0.1 mL/10 g of the mouse’s weight.

### 3.3. Acute Toxicity

Acute toxicity was estimated by calculating the 50% lethal dose (LD_50_) in mice by the intraperitoneal (i.p.) route. Animals were treated with IM-3 at doses of 400, 800, 1200, 2000 and 3000 mg/kg and were observed every 24 h for a period of 72 h to check for any deaths and to determine the LD_50_ of IM-3 [33].

### 3.4. Behavioral Pharmacological Screening

The experimental protocol established by Almeida *et al.* [21] was employed to evaluate the effect of IM-3 on the central nervous system. In this preliminary method, mice received IM-3 at doses of 50, 100 and 200 mg/kg, and resultant behavioral alterations in comparison with the control group (which received only the vehicle) were recorded at 30, 60, 120 and 240 min after treatment.

### 3.5. Motor Incoordination (Rotarod Test)

The rotarod test measures the effect of muscle relaxation and motor incoordination produced by drugs. It consists of placing mice on a bar that rotates at a constant speed and determining their ability to remain on it [34].

Animals were pre-selected 24 h prior to the experiment, according to their ability to remain on the rotating bar (7 rpm) for at least 3 min in 3 attempts. On the day of the experiment, the pre-selected mice were divided into groups (*n* = 8), treated with vehicle or IM-3 (50, 100 or 200 mg/kg), and evaluated at 30, 60 and 120 min after treatment. The parameter evaluated was the time spent on the bar for a period of up to 3 min. Animals that fell could be put back on the bar for up to 3 attempts.

### 3.6. Anxiolytic Activity (Elevated Plus-Maze Test)

The elevated plus-maze test is a simple and useful assay to access anxiety-related behavior induced by pharmacological agents in rodents. It consists of an elevated maze with two open and two enclosed arms and is based on the aversion of rats and mice to high and open environments, which is reduced by treatment with anxiolytic drugs [35].

Mice were divided into five groups (*n* = 8) and treated with the vehicle, IM-3 (50, 100 or 200 mg/kg) or 0.5 mg/kg diazepam (standard drug). Thirty minutes after administration, animals were individually placed in the center of the elevated plus-maze, with the nose facing one of the open arms, and observed for 5 min to record the number of entries and the time spent in the open arms.

### 3.7. Antinociceptive Activity Evaluation of IM-3

#### 3.7.1. Acetic Acid-Induced Writhing Test

The acetic acid-induced writhing test is based on the irritation evoked by intraperitoneal injection of 0.85% acetic acid in mice, which involves the stimulation of nociceptors and consequent behavioral reactions. The nociceptive effect is characterized by abdominal contortions followed by hind leg extension. In general, drugs with analgesic properties reduce or even inhibit this behavior [36].

Mice were divided into five groups (*n* = 8) and treated with vehicle, IM-3 (50, 100 or 200 mg/kg) or 6 mg/kg morphine. Thirty minutes later, each animal received 0.85% acetic acid and was placed in individual polyethylene boxes to record the latency to the first contortion and the number of writhes from 5 to 15 min after acetic acid injection

#### 3.7.2. Formalin Test

Mice were divided into five groups (*n* = 8) and treated with the vehicle, IM-3 (50, 100 or 200 mg/kg) or 10 mg/kg morphine (standard drug). After thirty minutes, 20 μL of 2.5% formalin were injected into the intraplantar region of the right hind paw of the mice. Afterwards, these animals were individually put in observation boxes, and the paw licking time was recorded for five minutes (first phase). After 10 min, the same parameter was determined for 15 min (second phase) [30,37].

#### 3.7.3. Hot Plate Test

This test consists of quantifying the reaction time of the animal to thermal stimulus, to evaluate analgesic activity mediated by central mechanisms [38,39].

Animals were subjected to a previous selection (without drug administration) and were considered suitable if they responded (tried to jump or lick the hind paws) for up to 15 s after being put on the hot plate (basal reading), which was set at 52 °C.

Mice were divided into five groups (*n* = 8) and treated with vehicle, IM-3 (50, 100 or 200 mg/kg) or 10 mg/kg morphine (standard drug). Thirty minutes after treatment, the animals were individually put on the hot plate, and the latency of response was recorded as the indication of antinociceptive action. This procedure was repeated at 60 and 120 min after administration. The animals stayed on the plate for a maximum of 30 s, to avoid tissue damage [22].

### 3.8. Statistical Analysis

The results presented were obtained using ANOVA, followed by Dunnett’s test. LD_50_ was calculated by non-linear regression. Data were analyzed using the GraphPad Prism program version 4.02, and the values obtained, except LD_50_, were expressed as the mean ± standard error of mean. *p* < 0.05 was considered significant.

## 4. Conclusions

Taken together, the results obtained in this work strongly suggest that the synthetic compound, IM-3, at the doses tested and by the intraperitoneal route, stimulates behavioral alterations in mice like CNS depressant drugs and reduces nociception, possibly through anti-inflammatory pathways. Further studies with appropriate pharmacological tools are necessary to elucidate the mechanisms involved.
